# Effect of different feeding methods and gut microbiota on premature infants and clinical outcomes

**DOI:** 10.3389/fnut.2022.888304

**Published:** 2022-08-01

**Authors:** Manman Liu, Cheng Chen, Songhao Kang, Jung-il Kwon, Juan Jin, Huilian Che

**Affiliations:** ^1^College of Food Science and Nutritional Engineering, China Agricultural University, Beijing, China; ^2^College of Engineering, China Agricultural University, Beijing, China; ^3^Maeil Innovation Center, Maeil Dairies Co., Ltd., Seoul, South Korea

**Keywords:** premature infants, breast milk, formula milk powder, Intestinal microecology, clinical outcomes

## Abstract

Premature infants require special care, and clinical feeding methods for this patient group are generally divided into breastfeeding and formula milk. This retrospective study investigated the effects of these two feeding methods on premature infants admitted to the neonatal intensive care unit between 2017 and 2018. Data regarding the duration of complete enteral feeding, weight gain, and postnatal infections were collected, categorized, and compared. Pearson’s correlation coefficient was used to determine the correlation between the intestinal flora and clinical outcomes. Results revealed no differences between the two feeding methods, and neither had significant effects on clinical indicators in premature infants, although the gut microbiota may be an important factor influencing many clinical indicators. Results of this study suggest an important role for the gut microbiota in the care of premature infants and provide a basis for promoting the healthy development of this patient population.

## Introduction

Nutrition in early life is a critical factor for neonatal growth and long-term health (1). Premature infants born before 37 weeks gestation and with lower weight and/or defective organ function account for 10% of births worldwide (2). Premature infants miss the expected intrauterine growth and accretion of nutrients in the third trimester, thus making the management of nutritional intake in premature infants a significant clinical challenge despite extensive study (3). Breast milk (BM) and specialized formula milk (FM) powder are the most common clinical feeding methods for premature infants (4). BM is considered to be the best source of nutrition for infants (5) and is superior in terms of regular gastrointestinal maturation, lower feeding intolerance, reducing the risk for necrotizing enterocolitis (NEC) and infection, and ameliorating long-term neurodevelopment (6). However, specialized FM powder represents a primary and appropriate alternative when the mother’s own milk is not available. To supply the nutrient requirements that enable infants to grow at the same rate as the fetus, a special FM powder for premature infants is often designed to boost energy, protein, and micronutrients (7). Studies have demonstrated that premature infants who receive FM exhibit faster growth, including weight, length, and head circumference, than those fed human milk, although with an increased risk for feeding intolerance and NEC (8).

In the mother’s body, in which the uterine environment is essentially sterile, the fetus is not exposed to microorganisms. During and after birth, however, they are exposed to the environment of the birth canal and surrounding microorganisms, causing the intestines to quickly colonize a wide variety of bacteria (9). It has been found that the composition of the intestinal flora is related to the body’s energy and nutrient intake (10, 11), which are pivotal factors for premature infants to risk off as soon as possible. Preterm infants, especially very-low-birth-weight infants, are susceptible to imbalances in the gut microbiota due to gut immaturity (12). The gut microbiome is directly affected by different feeding methods and affects physiological development, with long-term effects on the health of premature infants (13, 14). Infants fed BM exhibit greater initial bacterial diversity and a more gradual acquisition of variety than FM-fed infants (15). An ordered succession of microbial phylotypes has been observed in BM-fed infants; however, this succession appears to be disrupted in FM-fed infants. Studies involving full-term infants have found that BM-fed infants have more bifidobacteria and lactic acid bacteria in their intestines, while FM-fed infants exhibit fewer strict anaerobic bacteria (16), suggesting that the intestinal microecology may be a factor affecting clinical outcomes in this patient population.

Some published studies have addressed the use of BM or FM powder for premature infant nutrition fortification (17, 18); however, how the gut flora affects the clinical outcome of premature infants fed using different methods remains unclear. As such, this study explored the correlation between intestinal flora and clinical outcomes of premature infants to support tailoring optimal clinical strategies for their growth and development.

## Materials and methods

### Study population

Data from 31 infants admitted to the neonatal intensive care unit (NICU) at Peking University Third Hospital (Beijing, China) between September 2017 and September 2018, were collected. The present study was approved by the Human Research Protection Office. Newborn infants with a gestational age within 32 weeks or birth weight <2.0 kg were eligible for inclusion in the study; those who had congenital malformations and a survival time <7 days were excluded. Umbilical vein catheterization was performed in all premature infants who were treated with protective ventilation strategies, nutritional support, and prevention of infections based on clinical medical practice. Informed consent was obtained from the parents or legal guardians of all subjects before enrollment.

### Feeding patterns

In accordance with previous methods (19), all premature infants started enteral nutrition (EN) within 36 h of birth and the proportion of enteral nutrition was gradually increased until complete EN according to standard feeding guidelines (20). Owing to the promotion of breastfeeding in China, all infants were prioritized for BM.

Preterm infant formula for initial EN was used in cases of insufficient maternal milk. When developing to a plateau of 10–20 ml per day, premature infants were given priority to breastfeeding for successive feeding or continued formula feeding if BM was not available. According to the proportion of FM powder intake, >50% were assigned to the FM group, while the others were assigned to the BM group.

### Definition of clinical outcomes

Weight gain, NEC, postnatal infections, duration of NICU hospitalization, complete EN, and parenteral nutrition (PN) were defined as the major clinical outcomes. The secondary outcomes included blood biochemistry and routine blood tests. The duration of NICU hospitalization, EN, and PN were calculated from the day of birth. Weight gain was defined as the mean daily weight change during the NICU stay. Umbilical vein catheterization was performed in all preterm infants, and peripherally inserted central catheterization was performed according to the development of preterm infants, which commonly contributes to PN. The internationally revised Bell-NEC classification standard (Supplementary Table 1) was used to evaluate the severity of neonatal NEC, and levels above IIA were defined as NEC (21). The duration of antibiotic use was defined as the total number of days that the infants received ≥1 antibiotic(s).

Pneumonia was clinically diagnosed in the presence of respiratory distress, except for other factors (such as wet lung, patent ductus arteriosus, neonatal respiratory distress syndrome), as well as significantly augmented leukocyte counts and C-reactive protein levels in the blood, accompanied by new infiltrates on chest radiographs and positive pneumonia bacterial culture (22).

### Statistical analysis

Data regarding major morbidities, including NEC, late-onset sepsis, and types of infections, were collected from the medical charts of each preterm infant. Categorical data were compared using a two-tailed unpaired Student’s *t*-test or the chi-squared test and presented as the mean ± standard deviation (*SD*). Differences with *p*< 0.05 were considered to be statistically significant. Pearson’s correlation coefficient was used to determine the correlation between the intestinal flora and clinical outcomes.

## Results

### Premature infant cohort

A total of 31 infants, with a birth weight between 820 and 2,010 g (mean, 1,439 g) and gestational age between 25 and 32 weeks (mean, 28 weeks) fulfilled the inclusion criteria. Sixteen (52%) infants received BM and 15 (48%) received FM. The groups had similar baseline characteristics (Table 1), with no differences in birth weight, gestational age, sex, or delivery mode.

**TABLE 1:** Baseline characteristics of premature infants.

	BM (*n* = 15)	FM (*n* = 16)	*P*-value
Weight (g)	1412 ± 353	1464 ± 461	0.738
Gestational age (weeks)	30.9 ± 2.0	31.7 ± 2.6	0.396
Male (%)	5 (33.3%)	8 (50%)	0.197
Cesarean section (%)	10 (66.7%)	11 (68.8%)	0.861
PROM (%)	3 (33.3%)	2 (12.5%)	0.571
Eclampsia (%)	5 (33.3%)	7 (43.8)	0.552
Steroid hormones (%)	13 (86.7%)	13 (81.3%)	0.682

### Major clinical outcomes

**FIGURE 1:**
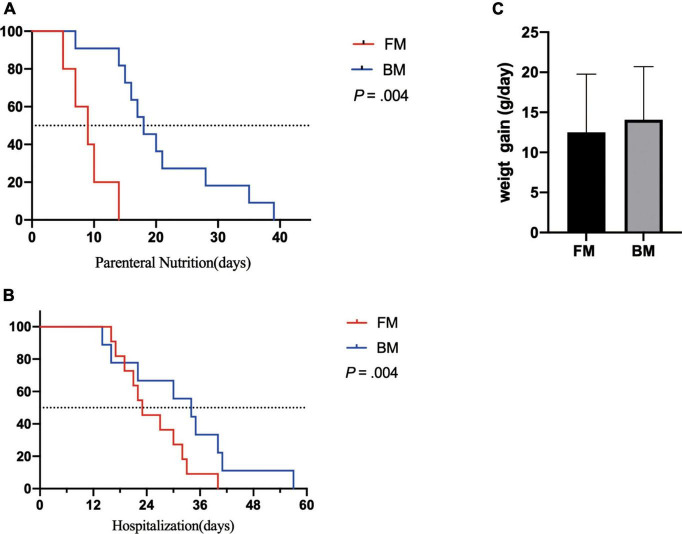
Major clinical outcomes between premature infants fed by breast milk and formula milk. **(A)** The days for parenteral nutrition between two groups. **(B)** The body weight gain per day (g/day) among infants. **(C)** The duration of hospitalization between two groups. *p* < 0.05 was considered a significant difference.

Both groups of premature infants started EN from the first day after birth and gradually increased the amount of milk from 1 ml fed every 8 h to achieve EN. Compared to premature infants who received BM, FM-fed infants had a significantly shorter duration to EN (*p* = 0.004). Most premature infants had a hospital stay of 14–57 days (Figure 1A). Similarly, the FM group (mean, 26 days) was discharged earlier than the BM group (mean, 36 days), as shown in Figure 1B. However, among the measures of average weight, the BM-fed preterm infants exhibited better weight gain than those who were FM-fed, as shown in Figure 1C.

Among the 31 premature infants, 15 acquired a total of 22 infections, of which pneumonia was the most common (Table 2). There were 11 pneumonia infections in the BM group and only 6 in the FM group. Among the 11 pneumonia infections in the BM group, 5 were caused by prenatal intrauterine infection, 1 was a ventilator pneumonia infection, and 1 was caused by a prenatal intrauterine infection in the FM powder group. Late-onset sepsis was the second-highest incidence of infectious diseases among premature infants in this study, which occurred only in the BM group. Among other types of infections (meningitis, urinary tract infection, orbital cellulite infection, and NEC), two groups did not occur. Jaundice symptoms during hospitalization did not demonstrate a significant difference, as indicated by direct bilirubin levels.

**TABLE 2:** Primary clinical outcome of premature infants.

	BM (*n* = 15)	FM (*n* = 16)	*P*-value
Average weight gain (g/day)	14.1 ± 6.4	12.5 ± 7.0	0.534
Parenteral nutrition duration (day)	7–32	6–34	0.131
Duration of ventilator (day)	12–39	3–67	0.460
Direct bilirubin	9.3 ± 4.3	11.0 ± 6.4	0.409
Number of infections	0–2	0–3	0.455
Pneumonia infections	11	6	0.265
Late-onset sepsis	4	0	0.322
Meningitis infections	0	0	–
Urinary tract infections	0	0	–
Orbital cellulite infections	0	0	–
Necrotizing enterocolitis	0	0	–
Duration of antibiotic use	0–17	0–40	0.063
Duration of gastric tube	20–51	4–77	0.197

### Secondary clinical outcomes in premature infants

Some important indicators of routine blood and blood biochemistry in clinical testing were selected as secondary clinical outcome indicators for premature infants. There were no significant differences in routine blood indicators between the two groups of premature infants. However, BM-fed infants exhibited higher levels of hemoglobin and platelets, which suggested that BM had a specific regulatory effect on the development of the immune system. Noticeable differences were observed in the levels of calcium, albumin (ALB), and alanine aminotransferase (ALT), which were significantly higher in BM-fed infants than in those who were FM-fed, as shown in Table 3.

**TABLE 3:** Secondary clinical outcomes in premature infants.

	BM (*n* = 15)	FM (*n* = 16)	*P*-value
WBC	16.7 ± 6.3	15.2 ± 6.1	0.519
RBC	4.96 ± 0.76	5.30 ± 0.78	0.250
HGB	205 ± 76	185 ± 26	0.335
PLT	401 ± 119	356 ± 91	0.261
HCT	55.1 ± 7.6	57.1 ± 9.4	0.517
MCV	114 ± 6.6	111 ± 6.7	0.145
MCHC	350 ± 12.2	341 ± 14.3	0.084
NEUTP	62.1 ± 12.1	54.1 ± 13.6	0.107
LYMPHP	53.2 ± 8.1	59.3 ± 8.7	0.058
MONOP	16.1 ± 3.9	14.3 ± 5.1	0.288
PCT	0.41 ± 0.10	0.36 ± 0.10	0.220
NA	142 ± 3.0	142 ± 4.6	0.717
K	5.52 ± 0.45	5.43 ± 0.96	0.762
CL	110 ± 2.3	112 ± 3.5	0.052
MG	1.00 ± 0.28	095 ± 0.22	0.590
CA	2.55 ± 0.32	2.28 ± 0.32	0.030
PHOS	2.25 ± 0.23	2.13 ± 0.35	0.268
CO_2_	24.6 ± 2.0	23.0 ± 5.3	0.320
ALT	13.4 ± 7.3	7.25 ± 2.9	0.005
AST	67.3 ± 52.9	53.9 ± 27.7	0.398
TP	50.9 ± 5.5	48.4 ± 4.6	0.185
ALB	33.8 ± 3.0	31.7 ± 1.5	0.022
RUN	8.16 ± 3.8	6.26 ± 2.1	0.108
CREA	84.1 ± 10.6	87.9 ± 10.9	0.342
URIC	480 ± 117	505 ± 97	0.531

However, owing to the difference between premature and normal infants, the reference ranges of various indicators have not yet been determined. Therefore, nutritional indicators for premature infants should be further explored.

### Correlation analysis of gut microbiota and clinical indicators

**FIGURE 2:**
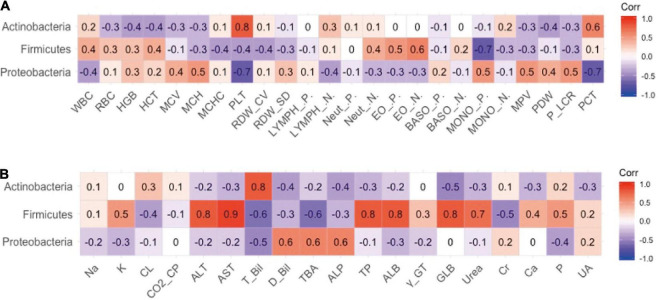
The relevance between phylum level of intestinal flora and secondary clinical outcomes. **(A)** The Pearson correlation coefficient of phylum level of intestinal flora and blood routine indicators. **(B)** The Pearson correlation coefficient of phylum level of intestinal flora and blood biochemistry indicators.

The authors previously explored different effects of FM and BM on the development of intestinal microecology in premature infants (19). The results revealed that BM feeding increased alpha diversity of the intestinal flora. Therefore, the relevance of different intestinal flora in clinical outcomes was explored. The analysis results of the phylum level of bacteria and routine blood tests are shown in Figure 2. The results indicated that the platelet (PLT) index was strongly correlated with the gut microbiota, and the abundance of proteobacteria was negatively correlated with the plateletcrit (PCT) and PLT. The phylum Actinobacteria was positively correlated with PCT and PLT levels. There appeared to be a negative co-variation between the Firmicutes phylum and monocyte percentage but was positively correlated with the number of eosinophil granulocytes. As shown in Figure 2B, the analysis revealed a significant association between the abundance of Firmicutes and some biochemical markers, in which a negative correlation existed between Firmicutes and total bilirubin (TBIL) and total bile acid (TBA) and a positive correlation with ALT, aspartate aminotransferase (AST), total protein, and ALB. It has been speculated that Firmicutes appear to affect the liver function of preterm infants. The phylum Actinobacteria is intimately associated with TBIL. There was a positive correlation between proteobacteria and alkaline phosphatase (ALP), TBA, and direct bilirubin.

**FIGURE 3:**
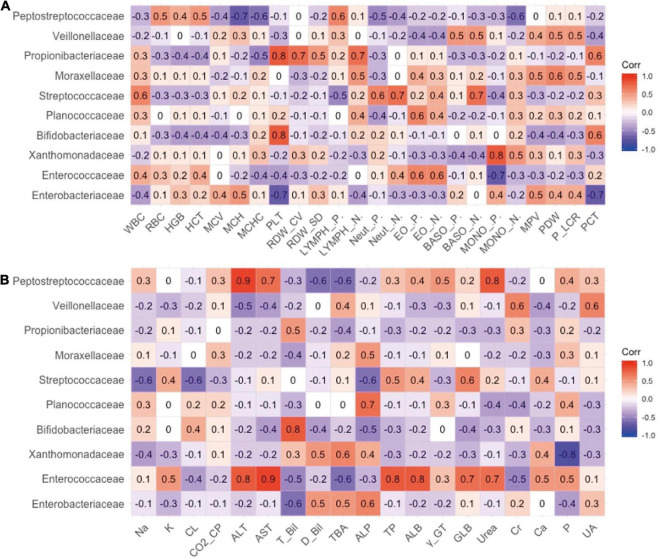
The relevance between the family level of intestinal flora and secondary clinical outcomes. **(A)** The Pearson correlation coefficient of the family level of intestinal flora and blood routine indicators. **(B)** The Pearson correlation coefficient of the family level of intestinal flora and blood biochemistry indicators.

The relationship between the gut microbiota and blood indices at the family level was also explored. As shown in Figure 3A, *Bifidobacteriaceae* and *Propionibacteriaceae* largely explained the correlation between Actinobacteria and PLT, and *Enterobacteriaceae* largely contributed to the negative correlation between Proteobacteria and PLT indicators. In addition, *Xanthomonadaceae* in Proteobacteria exhibited a significant positive correlation with monocytes, which may represent pathogens.

As shown in Figure 3B, *Enterococcaceae* may best contribute to the blood biochemical indicators. *Enterococcaceae* and *Peptostreptococcaceae* were the main factors that caused the increase in ALT, AST, and urea levels (Figure 3B). *Bifidobacteriaceae* in Actinomycetes contributed to the increase in TBIL.

## Discussion

Current research suggests that BM has the properties of fighting infection, stimulating the development of the immune system, and promoting tolerance and the anti-inflammatory response of the intestinal mucosal immune system after being challenged by bacterial pathogens (23). Many studies have investigated the clinical outcomes of FM and BM among term infants; however, few have involved preterm infants. In animal studies, formula-fed to premature piglets for only a few hours was sufficient to trigger an inflammatory response that was not suppressed by subsequent breastfeeding (24). Therefore, this study focused on the clinical outcomes of premature infants who consumed FM or BM.

The earliest multicenter randomized controlled study to investigate the clinical outcomes of preterm infants receiving BM of FM found that formula feeding significantly increased the probability of preterm infants experiencing NEC, sepsis, and other diseases, and the mortality of preterm infants was higher (25). Unlike previous studies, no significant differences were found between the two groups of premature infants in the relevant indicators of clinical infection in this study. In the bacterial culture test of preterm infants’ beds in this study, pathogens were detected in only a few beds, while not detected in those of most preterm infants, which may be attributed to environmental differences in the hospital. Furthermore, it has been reported that preterm infants with a gestational age <28 weeks were more susceptible to infectious diseases (26). All preterm infants in this study were 28–32 weeks old, which may be one explanation for the absence of infectious diseases.

In the case of poor health, FM powder lacks antimicrobial peptides, immunoglobulins, and other substances (27), which predisposed premature infants to the FM group. In contrast to previous studies, we found that FM-fed preterm infants achieved complete EN and were discharged from the hospital earlier than breastfed preterm infants. This may be attributed to the comprehensive nutritional composition of the formula, which enables it to be better absorbed. Among the secondary clinical outcomes of premature infants, we observed that blood calcium and albumin levels of premature infants in the BM group were significantly higher than those in the FM group, which may be related to the higher nutritional utilization of BM. Human milk is the preferred source of EN, in addition to nutrients, which contain components with a positive effect on tolerance to EN (28). However, human milk does not contain sufficient energy and the essential nutrients to meet the high requirements of premature infants, which is why premature infants exhibit a slower growth rate than FM-fed infants (29).

Feeding pattern is known to strongly influence the composition of the gut microbiota (30). In one study, the consecutive appearance of *Bacillales*, *Lactobacillales*, *Enterobacteriales*, *Clostridiales*, and *Bifidobacteriales* was found in infants fed with maternal breasts, while formula-fed infants experienced a longer persistence of *Bacillales* and *Lactobacillales* (15). This discrepancy was probably due to the relationship between the bacterial populations in human milk and the microbiota harboring the host gut. Therefore, the gut microbiota of the two groups of premature infants was also analyzed. By analyzing the correlation between gut microbiota and clinical indicators, we found that the abundance of Firmicutes was positively correlated with the levels of ALB, total protein, globulin, and uric acid and that there was a significantly favorable relationship between the abundance of Actinomycetes and TBA. Previous studies have reported that obese patients exhibit a predominance of Firmicutes bacteria (31, 32). It is speculated that Firmicutes bacteria are involved in intestinal energy absorption (33). In this study, Firmicutes bacteria were positively associated with multiple clinical nutritional indicators, which may also be caused by Firmicutes bacteria promoting energy absorption by the body. *Bifidobacteria* were positively related to bile acid and, due to a large amount of *Bifidobacteria* in BM, this result indicated that *Bifidobacteria* may be one of the causes of BM-induced jaundice; however, follow-up trials are still needed to confirm this.

The premature infants included in this study were all from one of the highest-ranking hospitals in China, ensuring that all enrolled children received appropriate treatment. A random observational method was used in this study, and factors, such as poor quality and bias, may have affected the research results. Due to the promotion of breastfeeding in China, few mothers are willing to feed their children with FM; therefore, the grouping of this study is based on the actual intake of FM and BM for premature infants. Because BM donation is not widely received in China and because of the lack of BM for their mothers in the first few days after birth, FM was used for the first few days of feeding in our clinical treatment. During stabilization, preterm infants were subjected to breastfeeding or mixed feeding according to their BM level. The lack of strict grouping was also one of the limitations of this study. However, this study endeavored to conduct strict mass observations on the basis of fulfilling ethical obligations, and the results are credible.

In the present study, we explored the effects of different feeding methods on the clinical outcomes of premature infants and the role of the gut microbiota. Limitations of this study include the relatively small sample size and its single-center design. Further data from premature infants of different ethnicities and regions are required.

## Conclusion

This study found no significant difference between BM and FM in the clinical outcomes of premature infants, and that the gut microbiota may be an important factor affecting some clinical indicators.

## Data availability statement

The datasets presented in this study can be found in online repositories. The names of the repository/repositories and accession number(s) can be found below: ENA; PRJEB34505.

## Ethics statement

The studies involving human participants were reviewed and approved by the Peking University Third Hospital Medical Science Research Ethics Committee. Written informed consent to participate in this study was provided by the participants’ legal guardian/next of kin.

## Author contributions

HC: conceptualization. CC and ML: data curation. ML, CC, and SK: formal analysis. HC, J-IK, and JJ: funding acquisition. ML, CC, and HC: writing–review and editing. All authors contributed to the article and approved the submitted version.
