# Supplementary material for: Effect of different feeding methods and gut microbiota on premature infants and clinical outcomes
Source: Front Nutr. 2022 Aug 1;9:888304. doi: 10.3389/fnut.2022.888304 (PMC9376281; doi:10.3389/fnut.2022.888304)
Supplement: Supplementary file 1 [file Table_1.pdf]

Supplementary Table 1 Bell-NEC classification standard

Stage	Systemic signs	Abdominal signs	Radiographic signs
IA Suspected	Temperature instability, apnea, bradycardia, lethargy	Gastric retention, abdominal distention, emesis, heme-positive stool	Normal or intestinal dilatation, mild ileus
IB---Suspected	Same as above	Grossly bloody stool	Same as above
IIA--Definite, mildly ill	Same as above	Same as above, plus absent bowel sounds with or without abdominal tenderness	Intestinal dilation, ileus, pneumatosis intestinalis
IIB Definite, moderately ill	Same as above, plus mild metabolic acidosis and thrombocytopenia	Same as above, plus absent bowel sounds, definite tenderness, with or without abdominal cellulitis or right lower quadrant mass	Same as IIA, plus ascites
IIIA Advanced, severely ill, intact bowel	Same as IIB, plus hypotension, bradycardia, severe apnea, combined respiratory and metabolic acidosis, DIC and neutropenia	Same as above, plus signs of peritonitis, marked tenderness, and abdominal distention	Same as IIA, plus ascites
IIIB Advanced, severely ill, perforated bowel	Same as IIIA	Same as IIIA	Same as above, plus pneumoperitoneum

DIC: disseminated intravascular coagulation

NPO: “nil per os” or nothing by mouth
